# Report of the General Council

**Published:** 1920-05-08

**Authors:** 


**King Edward's Hospital Fund for London**—(*continued*).

### REPORT OF THE GENERAL COUNCIL.

### A Landmark in the Fund's History.

1. The appointment of His Royal Highness the Prince of Wales as President of the Fund will make the year 1919 a landmark in its history. His Royal Highness presided over the annual meeting of the General Council in April, and in December over the distribution meeting. It is a source of great gratification to the members of the General Council that the chair occupied with such benefit to the Fund by His late Majesty King Edward VII. from 1897 to 1901 and His present Majesty King George V. from 1901 to 1910 is once more occupied by the heir to the throne.

2. Before leaving for Canada and the United States on August 5, His Royal Highness appointed the Marquess of Cambridge, the Earl of Iveagh, and the Right Hon. James W. Lowther, Speaker of the House of Commons, a Committee to exercise the powers of President during his absence from the United Kingdom, which lasted till December 1. The Council are greatly indebted to this Committee for their services.

The Year's Total Receipts.

3. The total receipts for the year 1919 were £201,477 0s. 9d., of which £8,422 2s. 2d. were contributions to capital, and £193,054 18s. 7d. receipts on general account.

(*Continued on page* 151.)

### KING EDWARD'S HOSPITAL FUND FOR LONDON.

(*Continued from p. 138.*)

4. General Fund receipts were made up as follow :—

| | | | |
|---|---|---|---|
| Annual subscriptions ... ... | £25,199 | 13 | 2 |
| Donations ... ... ... ... ... | 7,143 | 7 | 3 |
| League of Mercy ... ... ... | 17,000 | 0 | 0 |
| Legacies ... ... ... ... ... | 21,976 | 2 | 8 |
| Dividends and interest from investments ... ... ... | 116,535 | 15 | 6 |
| Trustees of the Bawden Fund ... | 200 | 0 | 0 |
| | 188,054 | 18 | 7 |
| Estate of Samuel Lewis, decd. ... | 5,000 | 0 | 0 |
| Making a total of ... ... | 193,054 | 18 | 7 |

The Exceptional Increase in Distribution.

5. The amount distributed, which had reached £200,000 for the first time in 1918, was increased to £230,000, chiefly in order to assist the hospitals in meeting the expenditure rendered necessary by the postponement of renewals and repairs during the war. The increase was thus an exceptional one made for a special purpose which will not recur.

6. The needs of the hospitals for ordinary maintenance purposes, however, have continued to show a rapid increase. According to the figures published in the Fund's Statistical Report, the total ordinary expenditure of the London hospitals in 1918 was £1,946,155, as against £1,724,288 in 1917; and the excess over 1913, which in 1917 was £580,500, amounted in 1918 to £803,217. Out of a total of 15,963 beds available in 1918 the average number in daily occupation by naval and military patients was 3,685, or 29 more than in 1917. The sum of £334,747 was received from the authorities towards the treatment of these naval and military patients, as against £238,960 in 1917. The balance, amounting to £468,470, represents the additional ordinary expenditure which fell on the funds of the voluntary hospitals of London during 1918, an increase of nearly 41 per cent. on the expenditure in 1913. A large part of this increased expenditure is due to the rise in prices, the development of expensive treatments and the provision of additional beds, and will therefore give the hospitals a claim to larger permanent support from the public.

Need for a Larger Income.

7. As will be obvious from the figures given in paragraph 4, the exceptional increase in the distribution was only rendered possible by drawing on the reserves which resulted from exceptional legacies and donations received during the war. The amount actually required to make up £230,000, after allowing for the expenses of the year, was £46,030 19s. 2d. It is clear that the maintenance of the distribution even at £200,000 will require a considerably larger income than was received in 1919, unless the reserves are to be further depleted, while the exceptionally urgent needs of the hospitals at the present time render a substantial increase in the normal distribution more than ever desirable.

8. Of the amount distributed, £218,000 was given to London hospitals, and £12,000 to consumption sanatoria and convalescent homes taking London patients, as against £190,000 and £10,000 respectively in 1918.

9. The £218,000 granted by the Fund to London hospitals was applied as to £164,775 in aid of general maintenance, £9,850 to the reduction of debts on maintenance account, £12,025 towards improvement schemes already undertaken or in reduction of liabilities on such schemes, and £31,350 in aid of schemes which it is proposed to carry out in the near future. Grants for maintenance amounted to £22,450 more than in 1917, or £14,375 more than in 1918.

10. Of the £12,000 distributed by the Convalescent Homes Committee, £8,770 was allocated to consumption sanatoria and £3,230 to convalescent homes. The grants to sanatoria enabled 66 beds to be reserved for the use of patients in London hospitals as compared with 64 last year. The Convalescent Homes Committee comment on the shortage of sanatorium beds for women patients.

11. The total sum distributed amongst hospitals, convalescent homes, and consumption sanatoria during the last ten years (after deducting conditional grants to the extent of £2,500 that have been allowed to lapse) is £1,754,500. Since the foundation of the Fund a total amount of £2,888,416 8s. 5d. has been distributed. The average of the 23 distributions during the whole period has been £125,583 6s. 5d.

12. During the year the amount spent on the administration of the Fund was £4,085 17s. 9d., or £2 0s. 6d. per £100 of the total received, as compared with £3,672 19s. 2d., or £1 13s. 7d. per £100 in the previous year. The average yearly outlay in this respect for the whole period since the inauguration of the Fund has been £2,954, the corresponding percentage of the receipts being £1 5s. 5d., or less than 3¼d. in every £1 received.

Annual Subscriptions.

13. His Majesty the King, Patron of the Fund, has been graciously pleased to continue his annual subscription of £1,000. Her Majesty the Queen, Her Majesty Queen Alexandra, and His Royal Highness the Prince of Wales have also been pleased to subscribe generously to the Fund, as have also their Royal Highnesses Prince Albert, Princess Mary, and many other members of the Royal Family.

14. The Fund again received annual subscriptions of £5,000 from Mr. Walter Morrison, £4,000 from the Drapers' Company, £1,000 from the late Viscount Astor, and £1,000 from the Merchant Taylors' Company. The contributions also included annual subscriptions of £525 from Lloyds Bank, Limited, and of £500 from the Earl of Iveagh, from Sir John R. Ellerman, Bart. (in addition to a special donation of £500), from Mr. Robert Fleming, from Messrs. Morgan, Grenfell and Co., from Messrs. N. M. Rothschild and Sons, and from the family of the late Sir S. Neumann, Bart.; and donations of £1,000 from Sir Frederick Green (a member of the Distribution Committee), £699 10s. 9d. from "S.D.R.S.D.," £500 from the Clothworkers' Company and from the Japanese Explosives Co., Ltd., and £385 from the profits of the charity cricket match, Surrey *versus* Middlesex, played at the Oval in July.

15. The amount contributed by the League of Mercy was again increased to £17,000, or £1,000 more than in 1918. The total amount contributed to the Fund by the League since its foundation in 1899 is now £293,000, apart from the sums awarded by the League to institutions outside the area of the Fund's distribution. The Council are deeply grateful to the large number of voluntary workers to whose efforts the continued success of the League of Mercy has been due.

16. Legacies received during the year included a further £10,000 from the estate of the late Brigade-Surgeon John Law, on account of residue; £5,000 from the estate of the

**King Edward's Hospital Fund for London—(*continued*).**

late Mr. James B. Dugdale; £3,739 17s. 8d. from the estate of the late Sir Julius Charles Wernher, Bart., further on account of residue left to capital; £5,000 from the estate of the late Mr. Samuel Lewis, further on account of residue; £3,000 from the estate of the late Mr. William Arthur Sturdy; and £2,500 from the Executors of the late Mrs. Sarah Morris—"Mr. and Mrs. John Morris Memorial Fund"—in the exercise of their discretionary powers.

BRITISH RED CROSS SOCIETY'S SURPLUS FUNDS.

17. In August 1919 the British Red Cross Society and the Order of St. John of Jerusalem, through their Joint War Committee, asked the King's Fund to undertake the distribution of the surplus funds available for hospitals in the London area. The conditions of this distribution will be the same as those of the similar distribution made by the Joint Committee itself amongst hospitals outside London—that is, grants can be made only to hospitals which can and will treat ex-sailors or ex-soldiers, and only in aid of definite schemes of extension or improvement that will be matured (*i.e.*, finally settled in the form in which they are to be carried out when circumstances permit) by March 31, 1921.

The amount of surplus Red Cross funds thus entrusted to the King's Fund for allocation will be £250,000, and the special distribution is expected to take place about the middle of 1920.

THE EXAMINATION OF HOSPITAL EXTENSION SCHEMES.

18. The question of hospital extensions has for some time been engaging the special attention of the Distribution Committee. By the end of 1917 schemes had been notified which, if they were all carried out, would have the effect of adding 1,750 to the number of beds available in 1913. By the end of 1918 the number of additional beds provided and contemplated amounted to about 4,000, of which about 1,000 had already been opened since 1913, and the Distribution Committee had begun a detailed examination of all these schemes partly in connection with the pending special distribution of surplus Red Cross funds, and partly for the general purpose of securing that no scheme should be undertaken without due regard to financial practicability, and to the general effect of all the schemes taken together.

19. Every eligible hospital which applied for a grant was inspected and reported upon, as hitherto, by two of the Fund's visitors, one medical and the other lay. The inspection of consumption sanatoria, which had been prevented for three years by the restrictions on travelling, was resumed in 1919.

20. The Council regret to record the death of Mr. H. B. Irving, who had been a visitor for the Fund since 1917.

RETIREMENTS FROM THE GENERAL COUNCIL.

The Council have also to regret the retirement of the Right Hon. Thomas Burt through ill-health. He was one of their original members, having been appointed to the General Council in 1897. The Council also regret the resignation of Lord Knutsford.

21. The President of the Local Government Board, in pursuance of his powers under the Act, has again appointed Messrs. Deloitte, Plender, Griffiths and Co., chartered accountants, to audit the accounts of the Fund for the year. They have been the honorary auditors since January 1898, during which period they have rendered the Fund very valuable service

THE PRESS AND THE VOLUNTARY HOSPITALS.

22. The Council are under great obligations to the newspaper Press for the assistance they have rendered in the past, and continue to rely upon their kindly co-operation in obtaining for the Fund wider publicity and support. Their help is more than ever valuable at a time like the present, when hospital expenditure has enormously increased, when the subscribing power of many regular contributors has diminished, and when, therefore, it becomes more and more important that the special value of the work of the voluntary hospitals, and their claims to increased support, should be brought home to a wider public.

St. James's Palace, April 30, 1920.

